# MRI diagnose post-operative anastomotic leak in patients with rectal cancer: preliminary experience

**DOI:** 10.1186/s12893-022-01872-w

**Published:** 2022-12-11

**Authors:** Liang Yu, Guangliang Chen, Hua Wang, Xiaojie Wang, Zhifen Chen, Ying Huang, Pan Chi

**Affiliations:** 1 Department of Colorectal Surgery, Fujian Medical University Union Hospital, 29 Xinquan Road, Fuzhou, 350001 Fujian China; 2 Department of Radiology, Fujian Medical University Union Hospital, Fuzhou, Fujian China

**Keywords:** Magnetic resonance imaging, Rectal resection, Diagnosis, Anastomotic leakage

## Abstract

**Background:**

Anastomotic leakage (AL) is one of the most serious postoperative complications after colorectal anastomosis. This study aims to evaluate the feasibility and diagnostic accuracy of magnetic resonance imaging (MRI) in the early detection of AL in patients with clinically suspected AL after rectal anterior resection.

**Methods:**

This was a prospective study including patients who underwent anterior resection and postoperative MRI examination. AL was diagnosed by comprehensive indictors, which were mainly confirmed by clinical signs, symptoms, and retrograde contrast enema (RCE) radiography. The sensitivity, specificity, positive predictive value (PPV), and negative predictive value (NPV) of diagnosing AL with MRI were calculated.

**Results:**

In total, 347 patients received anterior resection for rectal cancer, and 28 patients were suspected to have AL. Finally, 23 patients were included and received MRI examination. The median time interval from surgery to MRI was 10 days (3–21 days). The median distance from anastomosis to anal verge was 4.0 cm (2.0–10 cm), and 11 patients underwent diverted ileostomy. Eighteen patients had an anastomotic leak, including one patient who had a pelvic abscess and five patients who had no evidence of AL in the MRI examination. The overall sensitivity and specificity were 94.4% (95% CI 70.6% to 99.7%) and 80% (95% CI 29.8% to 98.9%), respectively. The PPV was 0.94 (95% CI 0.71 to 0.99) and the NPV was 0.80 (95% CI 0.29 to 0.99). For patients who had anastomosis less than 5 cm, the diagnostic accuracy of MRI was 93.7% (15/16). T2-weighted imaging with fat suppression can effectively reveal the leak track.

**Conclusions:**

The accuracy of plain MRI examination in diagnosing AL was favorable for patients with a suspected AL. T2-weighted imaging with fat suppression was the best imaging modality to diagnose AL. A multicenter prospective study with more samples is needed to further determine the safety and feasibility of MRI in the diagnosis of AL.

## Introduction

ANastomotic leakage (AL) is one of the most serious postoperative complications after rectal resection. The frequency of AL is 1.5% to 15% according to the previous literature [1–3]. Most of the mild to moderate leaks are managed conservatively using antibiotics and image-guided drainage, and some severe cases of AL will result in emergent reoperation and a temporary diverting ostomy due to generalized peritonitis or severe sepsis [4]. These severe cases of AL are associated with an increased perioperative mortality rate [5], and a recent meta-analysis demonstrated that severe cases of AL increased the local recurrence rate and decreased long-term overall survival, cancer-specific survival, and disease-free survival [6].

It is imperative to identify AL as early as possible given the severity of the complications associated with AL. Actually, clinical symptoms and signs of AL are not typical, and AL may be confused with other less life-threatening postoperative complications [7, 8]. Therefore, various radiographic imaging modalities have been used to assist in the early detection of AL.

Water-soluble contrast enema (WSE) and computed tomography (CT) imaging are the most prominent methods used to detect AL; however, some studies have shown that WSE has a high false-negative rate, even up to 46%, in patients with AL [9, 10]. CT has become the preferred diagnosis modality because it can provide a more precise image of anastomosis and diagnose the specific AL presented as the abscess beside anastomosis. CT also has the advantage of detecting other adverse events, such as intra-abdominal abscesses [11, 12]; however, CT is only 68% sensitive for diagnosing AL according to a systematic literature review [13]. CT combined with retrograde contrast enema (RCE) was reported to increase the sensitivity (83%) and specificity (97%) of diagnosing AL [13]. However, nonstandardized dispensing of contrast medium at the site of the anastomosis will compromise the accuracy of CT imaging in detecting AL [11].

Magnetic resonance imaging (MRI) has an undisputed role in the evaluation of anal fistula based on a sensitivity of up to 100% and a specificity of 86% for visualization of the fistula track [14, 15]. Whether MRI can be used to diagnose AL given its remarkable performance in discovering anastomosis defects and the leak track remains unknown. This study aims to evaluate the feasibility and diagnostic accuracy of MRI in the detection of AL in patients with clinically suspected AL after rectal surgery.

## Materials and methods

### Study population

This was a prospective study that was conducted at the Fujian Medical University Union Hospital (FMUUH) from November 2020 to June 2021. In total, 347 patients received anterior resection for rectal cancer during this period, and 28 patients were suspected to have AL. Finally, 23 patients were included and received MRI examination after the remaining 5 patients refused to undergo MRI. Three patients received hand-sewn anastomoses because they had rectal cancer that was too low to receive intersphincteric resection (ISR). The pelvic drain was placed routinely in patients who received rectal anterior resection in our institution. Generally, the drain tube was removed 5–7 days after surgery.

Anastomotic leakage after anterior resection of the rectum was defined as an interaction between the intra- and extraluminal compartments due to a defect in the integrity of the intestinal wall at the anastomosis between the colon and rectum or the colon and anus according to the consensus of the International Study Group of Rectal Cancer (ISREC) [16], and a pelvic abscess in the proximity of the anastomosis was also defined as anastomotic leakage. The signs and symptoms that indicated AL included anastomotic defect found by digital rectal examination (DRE), emission of gas, pus, or feces from the pelvic drain, peritonitis, signs of rectovaginal fistula, prolonged intestinal paralysis, fever, leukocytosis, pain, tenderness, and abdominal distention. The severity grading of AL was defined according to ISREC [16]. Grade A is defined as AL requiring no active therapeutic intervention. Grade B leaks require active therapeutic intervention but are manageable without laparotomy. Grade C leaks require relaparotomy.

All patients received MRI examination at an appropriate time after AL was suspected. The baseline clinicopathological characteristics and the clinical data after surgery, such as laboratory findings, surgery reports, radiology reports, symptoms, and signs, were recorded. This retrospective study was approved by the institutional research ethics committee of FMUUH (2021KY136).

### MRI acquisition

MRI examinations were achieved using a whole-body 3T MRI system (Magnetom Prisma, Siemens Healthcare) using an 18-channel body matrix coil combined with a 32-channel spine matrix coil. The scan protocol comprised 2D sagittal T2WI FS and 3-dimensional (3D) axial T2WI FS with coronal and sagittal reformations. The MR parameters used in 2D sagittal T2WI FS were as follows: FOV of 20 × 20 mm, slice thickness of 2 mm, TR/TE of 7680/84 ms. In addition, 3D axial T2WI FS was acquired with the following parameters: FOV of 23 × 23 mm, TR/TE of 1700/100 ms, and slice thickness of 1 mm without spacing. All patients received MR examination without enhancement scan and contrast enema.

### MRI analysis

**Fig. 1:**
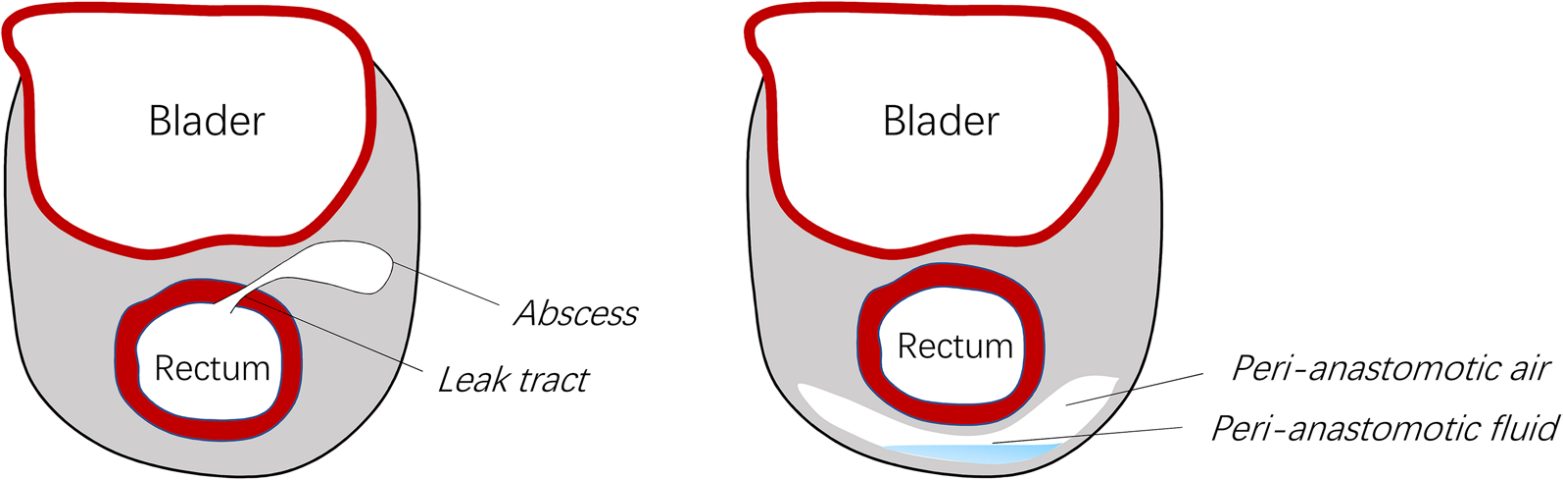
The schematic diagram of anastomostic leak

All MRI results for patients with suspected AL were independently evaluated by two experienced abdominal radiologists (GC & HW). The following features indicated AL: (1) presence of peri-anastomotic air and/or fluid; (2) disintegration of the anastomotic staple line; and (3) leakage of the tract from the anastomosis to the pelvic and/or adjacent organ (Fig. 1). MRI examinations were evaluated using a picture archiving and communication system (PACS, PLZ Healthcare) viewing station. The MRI results were recorded completely for further analysis.

### Statistical analysis

Demographic, disease, patient and radiology variable analyses were performed using descriptive statistics. The sensitivity, specificity, positive predictive value (PPV) and negative predictive value (NPV) were calculated. All analyses were performed with SPSS 23.0 for MAC (SPSS Inc., Chicago, IL, USA).

## Results

### Baseline characteristics

Twenty-three patients who underwent rectal resection with colorectal end-to-end anastomosis and were clinically suspected to have AL were included in the final analysis. Among them, there were 5 females and 18 males, and the median age was 58.1 (41–84) years old. The median distance from the inferior edge of the tumor to the anal verge was 8 cm (4–15 cm), and 13 patients received neoadjuvant therapy before surgery. The median distance from anastomosis to anal verge was 4.0 cm (2.0–10 cm), and 11 patients underwent diverted ileostomy. Anastomosis was performed by hand sewing in 3 patients and by stapling in 20 patients. Seventeen patients also received contrast enema (CE) radiography before or after MR examination at one- to two-day intervals. The clinical characteristics are shown in Tables 1 and 2.

**Table 1:** Patient demographics

Characteristic	Value (%)
Mean age (y ± SD) (range)	59.1 ± 11.7 (41–83)
Gender, n (%)	
Male	5 (21.7)
Female	18 (78.3)
Neoadjuvant chemotherapy	
Yes	13 (56.5)
No	10 (43.5)
Anastomotic methods	
Hand sewn	3 (13)
Stapler	20 (87)
Distance from anastomosis to anus, cm (mean ± SD) (range)	5.1 ± 2.5 (2–10)
Defunctioning stoma	
Yes	12 (52.2)
No	11 (47.8)
Index surgery to MRI, days (mean ± SD) (range)	10.3 (3–21)
Finally diagnosed anastomotic leakage	
Yes	18 (78.3)
No	5 (22.7)

**Table 2:** Individual patient characteristics

Patient	Gender	Age	nCRT^a^	Distance from anastomosis to anus (cm)	Anastomotic methods	Defunctioning stoma	Time to MR^b^ (days)	Fecal fluid from pelvic drainage tube	Increase in plasma inflammatory indicator^c^	Results of MR	Results of Contrast enema radiography
1	Male	52	Yes	4	Stapler	Yes	21	No	Yes	Pelvic abscess	Negative
2	Male	72	No	4	Stapler	No	9	No	Yes	Leak	Positive
3	Male	60	No	5	Stapler	No	11	No	No	Leak	Negative
4	Male	72	No	8	Stapler	No	5	Yes	Yes	Leak	Negative
5	Male	52	No	5	Stapler	No	5	Yes	Yes	Leak	Positive
6	Male	57	No	10	Stapler	No	7	Yes	Yes	No Leak	Positive
7	Female	60	Yes	3	Stapler	Yes	12	Yes	Yes	Leak	Positive
8	Female	58	No	2.5	Hand sewn	Yes	3	No	No	No Leak	Negative
9	Male	48	Yes	5	Stapler	No	5	Yes	Yes	Leak(suspect)	Negative
10	Male	43	Yes	5	Stapler	Yes	6	No	No	Leak	–
11	Male	43	Yes	3	Hand sewn	Yes	7	No	No	No leak	–
12	Male	63	Yes	8.5	Stapler	No	8	No	Yes	Leak (suspect)	Negative
13	Male	56	No	7	Stapler	No	10	Yes	Yes	Leak	Positive
14	Male	74	Yes	2	Stapler	Yes	11	No	Yes	No leak	–
15	Female	58	No	10	Stapler	No	11	No	Yes	No leak	Negative
16	Male	75	No	9	Stapler	No	13	Yes	No	Leak	Negative
17	Male	57	Yes	3	Stapler	Yes	14	No	No	Leak	–
18	Female	57	Yes	6	Stapler	No	15	No	Yes	Leak (suspect)	Negative
19	Male	41	Yes	4	Stapler	Yes	20	No	Yes	Leak	–
20	Male	84	No	4	Stapler	Yes	9	No	Yes	Leak (suspect)	Positive
21	Male	65	Yes	2.5	Stapler	Yes	11	Yes	Yes	Leak (suspect)	Positive
22	Male	42	Yes	4	Stapler	Yes	10	No	Yes	Leak	Positive
23	Female	70	Yes	2	Hand sewn	Yes	14	Yes	Yes	Leak	–

^a^Neoadjuvant chemoradiotherapy

^b^The time interval from operation to first MR examination

^c^The plasma inflammatory indicators included white blood cell count (WBC), C-reactive protein (CRP), interleukin-6 (IL-6), and procalcitonin (PCT)

### MR examination and outcome

**Fig. 2:**
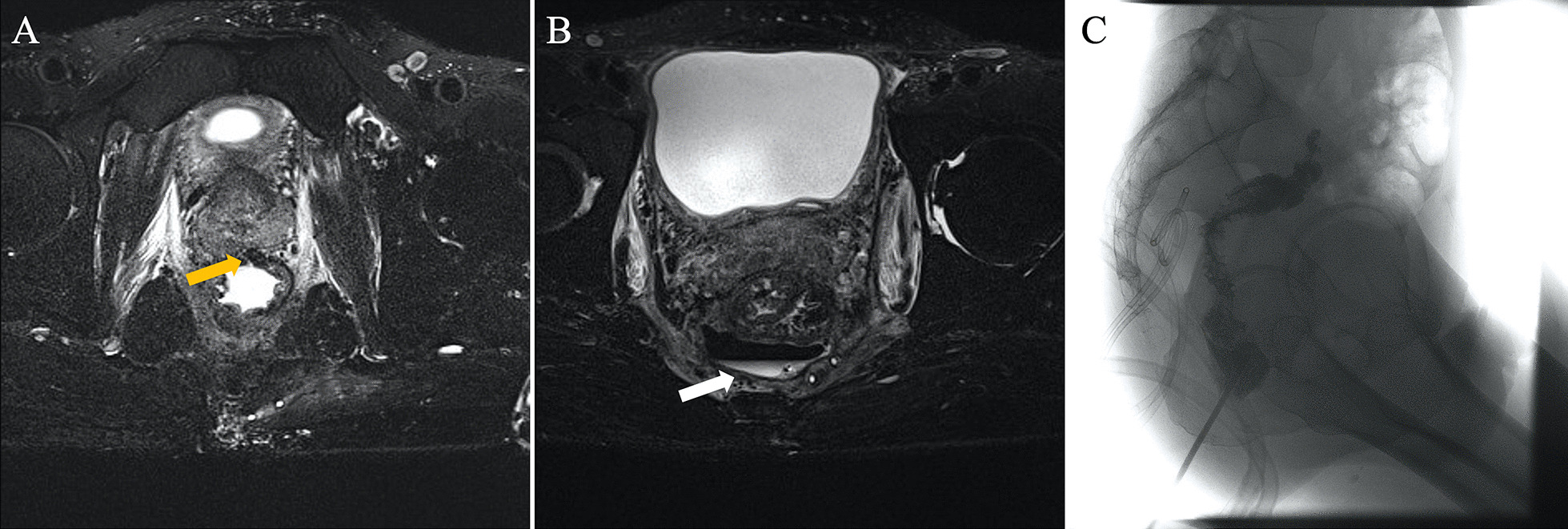
A suspected fistula tract (yellow arrow) at 12 o'clock can be seen on axial (fat suppression) T2-weighted MR images (**A**), suggesting anastomotic leakage in a 52-year-oldman; At a higher level image shows an abscess (white arrow) with a gas–liquid plane located in the presacral region (**B**). The above two signs were not found in barium enema (**C**)

**Fig. 3:**
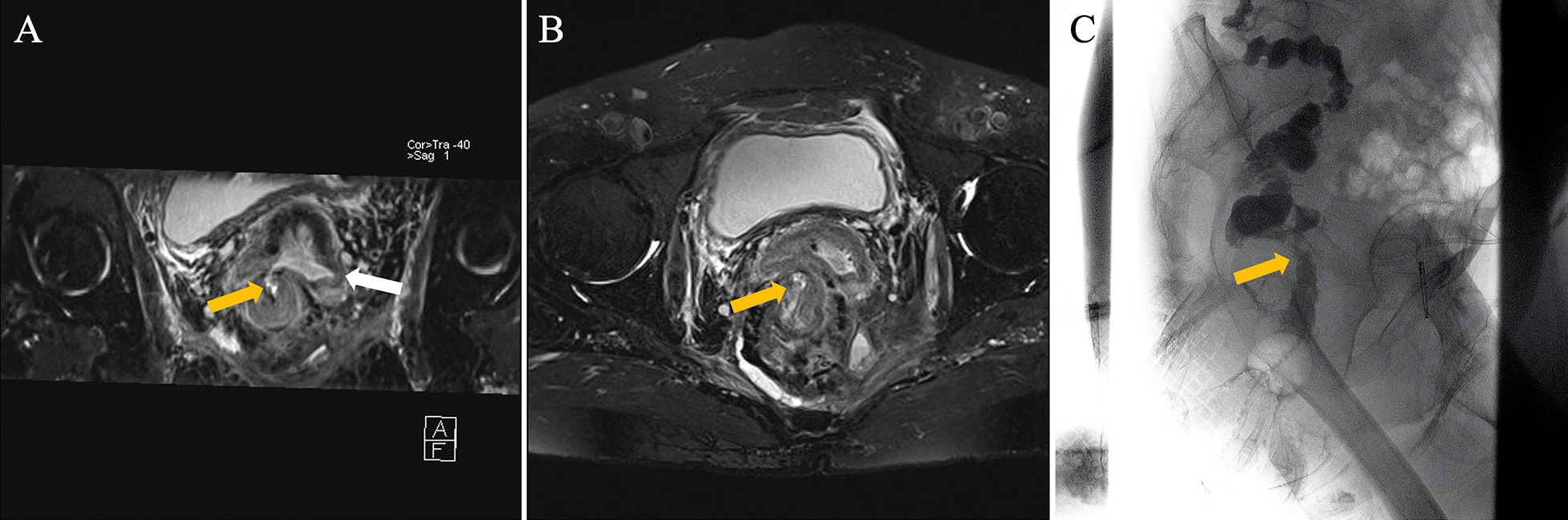
Oblique coronal (**A**) and axial (**B**) (fat suppression) T2-weighted MR images clearly show a fistula tract (yellow arrow) and an abscess (white arrow) in a 70-year-old woman. Barium enema (**C**) shows the unclear course of the fistula tract (yellow arrow)

All 23 patients signed the consent form and underwent MR examination after surgery. The median time interval from surgery to MR was 10 days (3–21 days). No adverse events caused by MRI were found during the study period. Eighteen patients were found to have an anastomotic leak, including one patient who had a pelvic abscess and five patients who had no evidence of AL on MRI examination. Among patients whose MRI indicated AL, three patients had no signs or symptoms of AL, but the bacterial culture of drainage fluid was positive, indicating a grade A leak. One patient whose anastomosis was located 10 cm from the anal verge had a negative MR result, but CE indicated an AL (Table 2). T2-weighted imaging with fat suppression effectively reveals the leak track, representing the best imaging method to estimate the AL according to our results (Figs. 2 and 3).

The overall sensitivity and specificity values were 94.4% (95% CI 70.6% to 99.7%) and 80% (95% CI 29.8% to 98.9%), respectively. The PPV was 0.94 (95% CI 0.71 to 0.99) and the NPV was 0.80 (95% CI 0.29 to 0.99). In total, 16 patients had an anastomosis smaller than 5 cm, and the diagnostic accuracy of MRI for these patients was 93.7% (15/16). For patients who received CE, eight patients had positive results, and nine patients had negative results. The sensitivity and specificity values were 37.5% (95% CI 16.3% to 64.1%) and 100% (95% CI 5.5% to 100%), respectively. The PPV was 1 (95% CI 0.52 to 1.00), and the NPV 0.09 (95% CI 0 to 0.43).

All patients were cured by conservative treatment, including pelvic drainage, antibiotics, and anal tube drainage.

## Discussion

AL following anterior resection is a severe complication that can increase morbidity and mortality. If AL is detected as early as possible, adverse events could potentially be reduced. Alves et al. found that the mortality rate increased from 0 to 18% if the diagnosis of a leak was made after the fifth postoperative day [17]. However, the signs and symptoms that could indicate AL were subtle in the majority of patients, leading to difficulty in diagnosing AL. Some leaks may take time to progress and will not present clinically until weeks after the operation. Previous studies showed that up to 42% of asymptomatic patients are diagnosed only upon readmission after initial hospital discharge and may even be diagnosed months later [18, 19]. In these cases, the early diagnosis of AL needs to be confirmed by imaging examination.

The best imaging method to detect AL has been debated in previous studies in past decades [18, 20, 21]. CE is a common method used to identify AL in clinical practice. However, the diagnostic efficacy of CE varies in different studies with accuracy values ranging from 5 to 80% [4], and the false-negative radiological result rate can reach up to 33% [22]. The sensitivity and specificity values were not ideal, and the NPV was only 0.2 in our study, which was consistent with a previous study.

CT is also an important imaging tool for assessing AL because it provides a more precise image of the anastomosis and peri-anastomotic structures compared with conventional radiology. A pelvic abscess close to the anastomosis is also considered AL according to the definition provided by ISREC, which indicates the diagnostic advantage of MRI and CT compared with CE. However, the sensitivity of CT scanning is unfavorable in some studies with values ranging from 14.8% to 68% [4, 12, 23]. The accuracy of CT in diagnosing AL is better when combined with CE. A retrospective study showed that contrast extravasation on CT was more often identified in the setting of a rectal anastomotic leak with a sensitivity and specificity of 83% and 97%, respectively [13]. However, the quality of CE influences the diagnostic accuracy of the CT scan. It is possible that the bacteria in the bowel lumen could be flushed into the pelvis by RCE, which would exacerbate the intra-abdominal infection. We have experienced two patients who developed generalized peritonitis after RCE in clinical practice, although previous studies rarely mentioned this adverse event of RCE. In addition, the accurate assessment of AL for lower anastomoses with or without enteric contrast remains more difficult.

MRI has been widely recognized as the gold standard imaging modality for the assessment of anal fistula due to its high accuracy in discriminating the fistula and surrounding structure [14, 24]. Although colorectal anastomosis leakage is different from anal fistula pathologically, we explored the diagnostic value of MRI in AL, and the results showed the high accuracy of MRI. Compared with CT scans and RCE, MRI has no radiation hazards and exhibit better repeatability. According to previous studies, when CE was applied, the contrast medium was instilled through a catheter placed in the rectum just below the anastomosis, and then 50–100 ml contrast medium was infused [22]. It is difficult to follow this protocol in patients with low anastomosis, which leads to the poorer accuracy of CE following CT in specific patients. In our study, there were 16 patients with anastomosis smaller than 5 cm, and the diagnosis accuracy of MR was 93.7% (15/16).

Guy et al. compared MRI-enema with fluoroscopic water-soluble contrast enema in the assessment of pelvic intestinal anastomotic integrity and found that MRI-enema offers a more detailed assessment of anastomotic leakage, providing additional pelvic information and an equivalent patient experience [25]. Although patients received MRI examination without enema in our study, the accuracy was satisfactory. The procedure without enema was simple. The preparation time required for an enema was eliminated, and the complications caused by retrograde enema, which causes patient discomfort, were avoided.

Furthermore, CT involves radiation, so the procedure cannot be performed again in a short period of time to evaluate the anastomosis after therapy. However, CT is quick and readily available. On the other hand, according to the results of our study, T2WI with fat suppression was considered to have the best diagnostic value. This finding indicated that patients with suspected AL could receive T2WI with fat suppression imaging to reduce the scan time. However, when considering the cost-effectiveness of health economics, the feasibility of MRI in the assessment of AL required further study.

Limitations of our study include the small sample size, which might influence the true diagnostic accuracy of MR. Second, this is a single-center prospective observational study with a short study period. Finally, the diagnosis of AL was confirmed by consensus between surgeons and radiologists based on clinical, laboratory, and radiological data in some patients with atypical signs and symptoms of AL due to the lack of a gold standard to diagnose AL.

In conclusion, the accuracy of plain MRI examination without CE in the diagnosis of AL was favorable for patients with suspected AL. A multicenter prospective study with more samples is needed to further estimate the safety and feasibility of MRI in diagnosing AL.

## Data Availability

The datasets used and/or analyzed during the current study are available from the corresponding author on reasonable request.

## Abbreviations

AL: Anastomotic leakage
MRI: Magnetic resonance imaging
PPV: Positive predictive values
NPV: Negative predictive values
WSE: Water-soluble contrast enema
CT: Computed tomography
RCE: Retrograde contrast enema
DRE: Digital rectal examination
ISGRC: International Study Group of Rectal Cancer
PACS: Picture archiving and communication system
CE: Contrast enema
